# Ensuring Reproducibility in Endovascular Aneurysm Repair: Clinical Impact of Prophylactic Branch Embolization in a Low-Volume Setting

**DOI:** 10.3400/avd.oa.26-00060

**Published:** 2026-09-10

**Authors:** Ryo Taguchi, Rin Itokawa, Kaito Muramatsu, Kazuo Ito, Norihiro Kondo

**Affiliations:** 1 Department of Cardiovascular Surgery, Aomori City Hospital, Aomori, Aomori, Japan; 2 Department of Thoracic and Cardiovascular Surgery, Hirosaki University Graduate School of Medicine, Hirosaki, Aomori, Japan; 3 Department of Surgery, Hirosaki Central Hospital, Hirosaki, Aomori, Japan

**Keywords:** endovascular aneurysm repair, proactive embolization, low-volume regional center, long-term sac behavior

## Abstract

**Objectives:**

Although prophylactic branch embolization is known to reduce Type II endoleak after endovascular aneurysm repair (EVAR), its reproducibility and clinical value in low-volume regional centers remain under-discussed. This study evaluated the efficacy of an anatomy-guided embolization strategy for improving long-term sac behavior.

**Methods:**

This retrospective, single-center cohort study included 132 patients who underwent elective EVAR. Prophylactic embolization (Coil+ group, n = 30) was performed based on standardized anatomical criteria, while 102 patients underwent standard EVAR (Coil− group). Preoperative anatomical risks and mid-term clinical outcomes, including sac expansion and re-intervention rates, were compared.

**Results:**

Preoperatively, the Coil+ group showed significantly higher anatomical risk than the Coil− group, including a higher prevalence of a patent inferior mesenteric artery (IMA) (90.0% vs. 69.6%; *p* = 0.031) and a greater mean IMA diameter (3.18 ± 0.73 mm vs. 2.76 ± 0.77 mm; *p* = 0.011). The Coil+ group achieved a 0% sac expansion rate at 24 months. Notably, the 5-year re-intervention rate was 0% (0/30) in the Coil+ group compared to 10.8% (11/102) in the Coil− group, representing a trend toward superior mid-term durability (*p* = 0.069).

**Conclusions:**

Standardized prophylactic branch embolization was associated with durable sac stability. This proactive approach may offer a rational pathway for optimizing long-term outcomes, even in low-volume centers.

## Introduction

Endovascular aneurysm repair (EVAR) has rapidly gained acceptance as the preferred treatment for abdominal aortic aneurysm (AAA),^1)^ owing to its reduced perioperative morbidity and mortality compared with open repair.^2)^ However, long-term durability remains a concern, particularly in relation to post-procedural endoleak.^3)^

Type II endoleak (T2EL) results from retrograde perfusion through branch vessels such as the inferior mesenteric artery (IMA) or lumbar arteries (LAs). This complication has historically been considered relatively benign and is often managed conservatively.^1,4)^ Nevertheless, accumulating evidence suggests that persistent T2EL is associated with aneurysm sac expansion and an increased risk of secondary intervention.^5)^ Consequently, the clinical relevance of T2EL has been re-evaluated in recent years.^6,7)^

In response to these concerns, prophylactic embolization of the IMA during EVAR has been advocated as a strategy to reduce the incidence of T2EL. Recent systematic reviews and meta-analyses have shown that IMA embolization significantly decreases the occurrence of T2EL.^3,8)^ Reflecting this evolving evidence, recent Japanese guidelines have recommended prophylactic embolization of the IMA in selected patients (Class IIa, Level of Evidence B).^9)^ Furthermore, several studies have suggested that embolization of multiple branch vessels, including LAs, may reduce the risk of persistent endoleak and influence sac behavior.^10,11)^

Despite these supportive data, routine embolization of abdominal side branches has not been widely adopted. This may be attributable to concerns regarding renal injury after EVAR,^12)^ including the potential impact of additional contrast exposure on renal function,^6)^ as well as prolonged operative and fluoroscopic times. A key concern relating to standardizing preemptive side branch embolization prior to EVAR is the complications related to the procedure.^13)^ Since EVAR itself carries a risk of renal injury, and renal injury can adversely affect long-term survival,^12)^ the clinical trade-off between the complexity of additional embolization and renal safety warrants careful investigation. Moreover, while embolization appears to be effective in reducing the incidence of T2EL, its impact on aneurysm sac regression and long-term aortic-related outcomes remains controversial. Some studies have suggested that a reduction in T2EL does not necessarily translate into meaningful improvements in sac remodeling or survival outcomes.^7,14)^ Thus, although prophylactic branch embolization is increasingly supported by the contemporary literature, its overall clinical benefit in real-world practice remains to be fully clarified. In particular, the balance between procedural complexity and long-term effectiveness warrants further evaluation.

The present study aimed to assess the clinical impact of prophylactic branch embolization during EVAR in a single-center cohort. Specifically, we evaluated the associations with: 1) the occurrence of T2EL; 2) aneurysm sac regression at 1 and 2 years; 3) long-term aortic-related events; and 4) changes in renal function. Through this analysis, we sought to further clarify the effectiveness and safety of branch embolization in routine clinical practice.

## Materials and Methods

### Study design and patient selection

We conducted a retrospective, single-center cohort study of patients who underwent elective EVAR for AAA between January 2015 and December 2025. The study was approved by the institutional review board of Aomori City Hospital (approval no. 2025-25). The requirement for informed consent was waived due to the retrospective nature of the study, with a provision for patient opt-out given on the hospital website. Prophylactic branch embolization (IMA and/or LAs) was introduced at our institution in October 2018. Patients were divided into 2 groups accordingly: the embolization group (Coil+), which included patients who underwent IMA and/or LA embolization from October 2018 onward, and the non-embolization group (Coil−), which comprised all patients treated before September 2018, as well as those treated without embolization after October 2018 (due to anatomical prerequisites, technical failure, or at the discretion of the surgeon).

### Exclusion criteria

Patients were excluded from the analysis if they met any of the following criteria: 1) urgent or ruptured AAA cases; 2) a follow-up period of less than 6 months; 3) incomplete clinical or radiological data; 4) performance of thoracic EVAR.

### Procedure and follow-up protocol

All EVAR procedures were performed using commercially available stent grafts in an angiography suite. Abdominal side branch embolization was adopted at our institution from October 2018. Candidates were selected based on the criteria described by Samura et al., including the presence of a patent IMA in conjunction with 1 or more of the following risk factors: a patent IMA ≥3 mm in diameter; LAs ≥2 mm in diameter; or an aortoiliac-type aneurysm.^15)^ In the embolization group, target branch vessels (IMA and/or LAs) were selectively embolized using metallic coils before or during stent-graft deployment. Follow-up involved clinical assessment and contrast-enhanced computed tomography (CT) at discharge, 6 months, 12 months, and annually thereafter. Renal function was assessed by measuring the estimated glomerular filtration rate (eGFR) preoperatively, at discharge, and during each follow-up visit.

### Evaluated endpoints and definitions

#### Occurrence of T2EL

The occurrence of T2EL was defined as the presence of any T2EL confirmed by contrast-enhanced CT during follow-up.

#### Aneurysm sac behavior

The maximum transverse diameter of the aneurysm sac was measured. Sac regression (shrinkage) was defined as a decrease of ≥5 mm, whereas sac expansion was defined as an increase of ≥5 mm compared with the first postoperative CT scan.

#### Long-term aortic-related events

Long-term aortic-related events included aneurysm-related death, aneurysm rupture, and the need for secondary re-intervention (e.g., additional embolization or open conversion).

#### Renal function change

The change in renal function was evaluated by the change in eGFR (ΔeGFR) from the preoperative baseline to the latest follow-up and the incidence of acute kidney injury.

### Statistical analysis

Continuous variables are expressed as the mean ± standard deviation or median with interquartile range and were compared using Student’s t-test or the Mann–Whitney U test, as appropriate. Categorical variables were compared using the chi-square test or Fisher’s exact test. Specifically, the distribution of stent-graft devices, including AFX (Endologix, Irvine, CA, USA), Endurant (Medtronic, Dublin, Ireland), and Excluder (Gore Medical, Flagstaff, AZ, USA), between the 2 groups was analyzed using the chi-squared test to evaluate potential selection bias. To account for the competing risk of mortality and differing follow-up lengths, a landmark analysis at 60 months was performed for re-intervention-free survival. Survival curves were constructed using the Kaplan–Meier method and compared using the log-rank test.

All statistical analyses were performed using R version 4.5.2 (R Foundation for Statistical Computing, Vienna, Austria). The primary packages used were survival, coxphf, lme4, and lmerTest. All statistical tests were 2-sided, with values of *p* <0.05 considered statistically significant. Missing data were handled by complete-case analysis for each endpoint-specific model, and no imputation was performed.

## Results

### Patient characteristics and device distribution

A total of 132 patients were analyzed, comprising 30 patients in the prophylactic branch embolization group (Coil+) and 102 patients in the non-embolization group (Coil−). Baseline characteristics are summarized in **Table 1**. The 2 groups were well balanced regarding age, sex, and comorbidities, including hypertension, diabetes, and smoking history. No significant differences between groups were observed in the use of antiplatelet or anticoagulant therapies. Notably, the distribution of stent-graft devices showed no significant difference between the Coil+ and Coil− groups: AFX (36.7% vs. 27.5%, respectively); Endurant (23.3% vs. 35.3%, respectively); and Excluder (40.0% vs. 37.3%, respectively) (*p* = 0.421). Preoperative body weight was significantly higher in the Coil+ group (68.2 ± 12.7 kg vs. 61.0 ± 12.1 kg, respectively; *p* = 0.006). Preoperative anatomical characteristics related to T2EL risk are summarized in **Table 1**. The Coil+ group exhibited a significantly higher prevalence of high-risk anatomical features compared to the Coil− group. Specifically, the Coil+ group showed a higher rate of patent IMAs (90.0% vs. 69.6%; *p* = 0.031) and a significantly higher proportion of patients with an IMA diameter ≥3.0 mm (73.3% vs. 44.1%; *p* = 0.007). Furthermore, the number of patent LAs was significantly greater in the Coil+ group (3.8 ± 1.4 vs. 3.0 ± 1.4, respectively; *p* = 0.013), as was the presence of LAs ≥2.0 mm in diameter (76.7% vs. 52.9%; *p* = 0.022). These findings indicate that branch embolization was selectively performed in patients with a higher anatomical predisposition toward T2EL. To clarify the adherence to our institutional protocol introduced in October 2018, among the 50 patients who met the inclusion criteria during the post-introduction period, 26 had a patent IMA ≥3.0 mm in diameter. Thirty-nine patients had 1 or more patent LAs measuring ≥2.0 mm in diameter. Of these 39 patients, 15 also had a patent IMA ≥3.0 mm, 18 had a patent IMA <3.0 mm, and 6 had an occluded IMA. Prophylactic embolization was attempted in 41 patients, yielding a protocol adherence rate of 82.0%. Regarding technical success, IMA embolization was attempted in 24 cases and was successful in 22 cases (91.7%), whereas LA embolization was attempted in 58 vessels and was successful in 49 vessels (84.5%). The primary reasons for not performing embolization after October 2018 were an acute iliac artery angle that precluded microcatheter access or severe calcification at the vessel ostium. Regarding the anatomical extent of the procedures in the Coil+ group, the mean number of embolized vessels per patient was 1.7 ± 0.7 (range, 1–4). Specifically, 14 patients (46.7%) underwent isolated IMA embolization, 13 patients (43.3%) underwent combined IMA and LA embolization, and 3 patients (10.0%) underwent isolated LA embolization.

**Table 1:** Patient background and baseline characteristics

Variables	Coil– (n = 102)	Coil+ (n = 30)	*p*-Value
Age (years)	75.5 ± 8.4	74.8 ± 7.9	0.692
Sex (male)	87 (85.3%)	26 (86.7%)	1
Body weight (kg)	61.0 ± 12.1	68.2 ± 12.7	0.006*
Hypertension	95 (93.1%)	29 (96.7%)	0.682
Hyperlipidemia	48 (47.1%)	14 (46.7%)	1
Diabetes mellitus	18 (17.6%)	5 (16.7%)	1
Smoking history	74 (72.5%)	17 (56.7%)	0.118
COPD	30 (29.4%)	10 (33.3%)	0.659
Ischemic heart disease	42 (41.2%)	11 (36.7%)	0.833
Atrial fibrillation	16 (15.7%)	4 (13.3%)	1
Antiplatelet therapy	57 (55.9%)	14 (46.7%)	0.41
Anticoagulant therapy	18 (17.6%)	4 (13.3%)	0.782
Preoperative aneurysm diameter (mm)	53.1 ± 12.7	49.7 ± 8.8	0.179
Preoperative eGFR (mL/min/1.73 m^2^)	58.8 ± 15.3	63.5 ± 18.1	0.167
Stent-graft device			0.421^‡^
AFX	28 (27.5%)	11 (36.7%)	0.366^†^
Endurant	36 (35.3%)	7 (23.3%)	0.271^†^
Excluder	38 (37.3%)	12 (40.0%)	0.832^†^
Preoperative anatomical risk factors			
Patent IMA	71 (69.6%)	27 (90.0%)	0.031*
IMA diameter (mm)	2.76 ± 0.77	3.18 ± 0.73	0.011*
IMA diameter ≥3.0 mm	45 (44.1%)	22 (73.3%)	0.007*
Number of patent LAs	3.0 ± 1.4	3.8 ± 1.4	0.013*
Number of LAs ≥2.0 mm	0.8 ± 0.9	1.2 ± 0.8	0.052
Presence of LAs ≥2.0 mm	54 (52.9%)	23 (76.7%)	0.022*

^†^Fisher’s exact test (each device vs. others).

^‡^Chi-squared test.

Continuous variables are expressed as the mean ± standard deviation. Categorical variables are expressed as the number and percentage.

COPD, chronic obstructive pulmonary disease; eGFR, estimated glomerular filtration rate; IMA, inferior mesenteric artery; LA, lumbar artery

Regarding non-T2ELs, a postoperative Type Ia endoleak occurred in 1 patient in the Coil− group and resolved spontaneously without secondary intervention. One patient in the Coil− group developed a late Type Ib endoleak at 5 years and underwent secondary intervention using an iliac branch endoprosthesis. Porosity-related Type IV endoleaks were noted in 2 patients (1 in each group) postoperatively, both resolving during follow-up. Crucially, no patients in the Coil+ group required secondary interventions for Type I or Type III endoleaks, confirming that the re-intervention events in the Coil+ group were strictly evaluated without confounding by non-T2EL complications.

### Perioperative and renal outcomes

As detailed in **Table 2**, the Coil+ group required a significantly higher volume of contrast medium (171.8 ± 80.5 mL vs. 114.9 ± 58.8 mL, respectively; *p* <0.001) and longer fluoroscopy time (65.3 ± 36.5 min vs. 51.3 ± 31.8 min, respectively; *p* = 0.025). However, no significant differences were seen in operative time (158.4 ± 69.2 min vs. 165.9 ± 62.3 min, respectively; *p* = 0.485), ΔeGFR (1.8 ± 6.3 mL/min/1.73 m^2^ vs. 1.8 ± 8.3 mL/min/1.73 m^2^, respectively; *p* = 0.727) or eGFR change rate (4.1 ± 14.5% vs. 5.0 ± 13.4%, respectively; *p* = 0.732).

**Table 2:** Perioperative and renal outcomes

Outcome	Coil– (n = 102)	Coil+ (n = 30)	*p*-Value
Perioperative data			
Operative time (min)	158.4 ± 69.2	165.9 ± 62.3	0.485
Contrast media volume (mL)	114.9 ± 58.8	171.8 ± 80.5	<0.001*
Fluoroscopy time (min)	51.3 ± 31.8	65.3 ± 36.5	0.025*
Anatomical extent of embolization			
IMA embolization alone, n (%)	–	14 (46.7%)	–
Combined IMA + LA embolization, n (%)	–	13 (43.3%)	–
LA embolization alone, n (%)	–	3 (10.0%)	–
Number of embolized vessels per patient	–	1.7 ± 0.7 (range 1–4)	
Renal function outcomes			
Change in eGFR (mL/min/1.73 m^2^)	1.8 ± 6.3	1.8 ± 8.3	0.727
eGFR change rate (%)	4.1 ± 14.5	5.0 ± 13.4	0.732
Endoleak and sac behavior			
Any Type II endoleak	45 (44.1%)	8 (26.7%)	0.095
Persistent Type II endoleak^†^	36 (35.3%)	7 (23.3%)	0.271
Aneurysm sac diameter change (mm)	−4.9 ± 10.2	−3.8 ± 6.6	0.826
Aneurysm sac shrinkage (≥5 mm)	44 (43.1%)	10 (33.3%)	0.401
Re-intervention and follow-up			
Overall secondary re-intervention	12 (11.8%)	1 (3.3%)	0.296
Secondary re-intervention within 5 years	11 (10.8%)	0 (0.0%)	0.069
Follow-up period (months)	52.9 ± 31.1	38.9 ± 21.7	0.033*

^†^Persistent Type II endoleak: observed at 6 months or later follow-up.

Continuous variables are expressed as the mean ± standard deviation. Categorical variables are expressed as the number and percentage.

IMA, inferior mesenteric artery; LA, lumbar artery; eGFR, estimated glomerular filtration rate

### Mid-term sac behavior and predictors of expansion (Table 3)

**Table 3:** Early follow-up outcomes (1 and 2 years)

Follow-up time	Parameter	Coil–	Coil+	*p*-Value
1 year	Type II endoleak	25/102 (24.5%)	4/30 (13.3%)	0.222
	Sac shrinkage (≥5 mm)	29/92 (31.5%)	7/28 (25.0%)	0.64
	Sac expansion (≥5 mm)	1/92 (1.1%)	0/28 (0.0%)	1
	Change in eGFR* (mL/min/1.73 m^2^)	−3.4 ± 7.3	−4.9 ± 9.5	0.784
2 year	Type II endoleak	17/102 (16.7%)	6/30 (20.0%)	0.784
	Sac shrinkage (≥5 mm)	31/84 (36.9%)	11/25 (44.0%)	0.64
	Sac expansion (≥5 mm)	11/84 (13.1%)	0/25 (0.0%)	0.065
	Change in eGFR* (mL/min/1.73 m^2^)	−6.4 ± 9.2	−5.0 ± 9.1	0.26

*Change in eGFR is expressed as the mean ± standard deviation.

eGFR, estimated glomerular filtration rate

As of the 2-year follow-up, the Coil+ group demonstrated a higher rate of sac shrinkage by >5 mm compared to the Coil− group (44.0% vs. 36.9%, respectively). Crucially, no cases of sac expansion by >5 mm were observed in the Coil+ group (0.0%), whereas 13.1% of patients (11/84) in the Coil− group experienced enlargement (*p* = 0.065). Multivariate logistic regression analysis (**Table 4**) identified T2EL at 2 years (odds ratio [OR] 9.49, 95% confidence interval [CI] 2.58–34.88; *p* = 0.001) and preoperative aneurysm diameter (OR 1.07, 95% CI 1.02–1.12; *p* = 0.005) as independent predictors of long-term sac expansion.

**Table 4:** Uni- and multivariate logistic regression analysis for long-term aneurysm sac expansion

Variable	Univariate OR (95% CI)	*p*-Value	Multivariate OR (95% CI)	*p*-Value
Type II endoleak at 2 years	7.77 (2.46–24.55)	0.001*	9.49 (2.58–34.88)	0.001*
Preoperative aneurysm diameter (per 1 mm)	1.05 (1.01–1.10)	0.007*	1.07 (1.02–1.12)	0.005*
Chronic obstructive pulmonary disease	0.14 (0.02–1.13)	0.065	0.18 (0.02–1.55)	0.118
Antithrombotic therapy	1.01 (0.34–3.02)	0.99	—	—
Branch embolization	0.83 (0.22–3.17)	0.789	—	—
Number of embolized vessels	0.79 (0.34–1.81)	0.571	—	—

Values of *p* <0.05 were considered statistically significant.

OR, odds ratio; CI, confidence interval

### Subgroup analysis in patients with anatomically high-risk features

To clarify the definitive clinical benefit of prophylactic embolization, a subgroup analysis was performed restricted to patients presenting with preoperative anatomically high-risk features (defined as IMA ≥3.0 mm or LA ≥2.0 mm; total n = 100), as summarized in **Table 5**. Within this high-risk subset, the Coil+ group achieved a 0% (0/25) sac expansion rate at the 2-year follow-up and a 0% secondary re-intervention rate at the 60-month landmark. In contrast, the high-risk Coil− group (n = 70) demonstrated an 11.4% (8/70) 5-year re-intervention rate.

**Table 5:** Subgroup analysis of clinical outcomes in anatomically high-risk patients

Outcome	Coil− (n = 70)	Coil+ (n = 30)	*p*-Value
2-Year Postoperative Outcomes			
Type II endoleak, n/N (%)	12/55 (21.8%)	5/25 (20.0%)	1.000
Aneurysm sac shrinkage (≥5 mm), n/N (%)	26/55 (47.3%)	11/25 (44.0%)	0.814
Aneurysm sac expansion (≥5 mm), n/N (%)	5/55 (9.1%)	0/25 (0.0%)	0.318
Long-Term Events (60-Month Landmark)			
Secondary re-intervention within 5 years, n/N (%)	8/70 (11.4%)	0/30 (0.0%)	0.101

CT data at 2 years were available for 80 patients.

Values are expressed as the number and percentage. N represents the number of patients with computed tomography data available from the 2-year follow-up. Values of *p* <0.05 were considered statistically significant (Fisher’s exact test).

### Freedom from aortic-related events

**Fig. 1:**
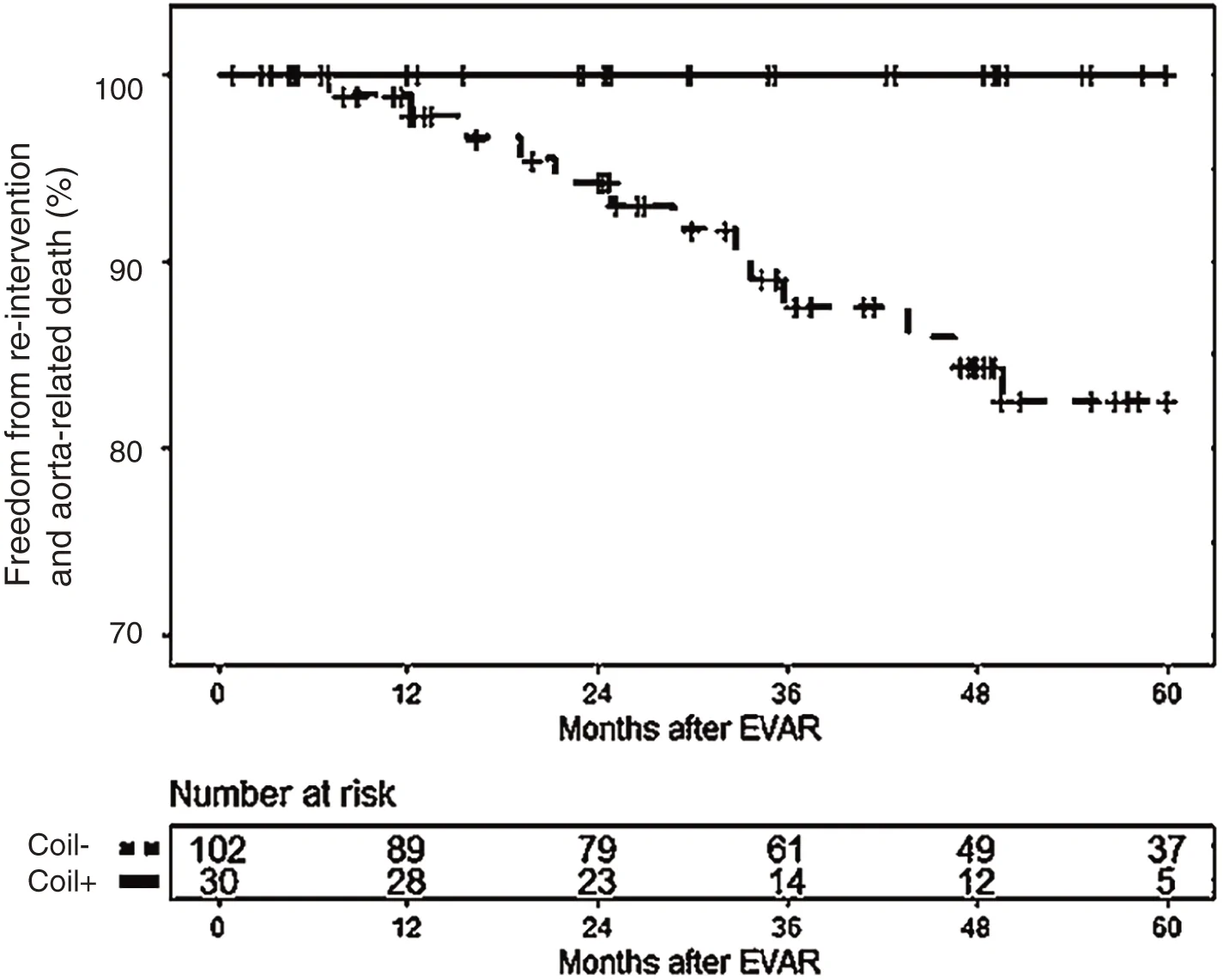
Cumulative Kaplan–Meier estimates of freedom from re-intervention and aorta-related death for patients undergoing EVAR with (Coil+: solid line) or without (Coil−: dashed line) IMA and/or LA embolization. EVAR, endovascular aneurysm repair; IMA, inferior mesenteric artery; LA, lumbar artery

Freedom from aortic-related events was defined as freedom from a composite of aneurysm rupture, aneurysm-related death (including sudden unexplained death), and secondary re-intervention. The 60-month landmark analysis showed that the Coil+ group achieved a 100% event-free rate at 60 months, with no instances of rupture or re-intervention. In contrast, the Coil− group had an event-free rate of 87.3%, with 13 recorded events (including 11 re-interventions and 1 event each of rupture and sudden unexplained death). Log-rank testing showed a strong trend toward improved long-term stability in the Coil+ group (*p* = 0.069) (**Fig. 1**). A single secondary re-intervention was performed 63 months postoperatively within the Coil+ group. This case, which had initially undergone isolated IMA embolization, required subsequent LA coil embolization due to LA-driven T2EL accompanied by aneurysm sac expansion.

## Discussion

The objective of infrarenal AAA repair is to prevent rupture and death. Successful aneurysm treatment prolongs life only if aneurysm rupture was destined to occur.^2)^ Accurate prediction of which aneurysms will rupture is not currently possible, although the risk increases with increasing aneurysm size.^1)^

In the present study, the Coil+ group demonstrated complete suppression of aneurysm sac expansion at 24 months and 100% freedom from aortic-related events at 60 months. These findings highlight the importance of achieving definitive mechanical exclusion of collateral inflow during the index procedure. Although the Society for Vascular Surgery (SVS) clinical practice guidelines recommend intervention when the sac grows by ≥5 mm,^1)^ Japanese guidelines highlight the lack of consensus regarding optimal treatment strategies for T2EL.^9)^ A recent investigation of large-scale data from Vaddavalli et al. demonstrated that secondary interventions for T2EL were not associated with improved survival, despite significantly higher re-intervention rates.^7)^ These findings suggest that a reactive strategy based on post hoc intervention may have a limited impact on long-term survival outcomes. Given that sac expansion has been associated with both late rupture and aneurysm-related death,^16)^ these insights support the potential value of proactive prevention of T2EL as a more effective pathway for optimizing long-term patient outcomes compared to reactive management of its consequences. From this perspective, a proactive strategy targeting anatomical risk factors at the time of EVAR seems likely to provide a more effective approach. Samura et al. identified objective predictors of persistent T2EL, including an IMA diameter ≥3.0 mm and an LA diameter ≥2.0 mm,^15)^ whereas Takeuchi et al. demonstrated in a randomized trial that pre-emptive embolization significantly reduced T2EL occurrence and improved long-term sac shrinkage.^17)^ The technical extent of embolization remains controversial.^18)^ While some studies suggest that IMA embolization alone may be sufficient,^3,8,19)^ others report that comprehensive embolization,^10,11,13)^ including of LAs, provides superior outcomes. Our findings support the clinical utility of tailored branch management based on objective anatomical risk factors rather than a rigid uniform strategy. Although the small sample size precluded statistically valid direct comparisons between isolated IMA embolization and combined IMA+LA embolization, the protocol successfully achieved sufficient mid-term sac stability by selectively addressing the LAs when high-risk features were present. Although 1 case in the Coil+ group eventually required secondary intervention for LA-driven T2EL at 63 months, this single case suggests that late LA-driven T2EL can still occur after prophylactic embolization and underscores the need for continued surveillance. Historically, T2EL has been regarded as a benign phenomenon often managed conservatively, but recent evidence has emphasized the necessity of risk stratification, as T2ELs driven by a well-developed IMA or multiple patent LAs carry a higher risk of persistent sac expansion. By mechanically neutralizing the most prominent collateral pathways during the index procedure, our proactive approach may shift the natural history of high-risk aneurysms, significantly delaying or preventing late sac enlargement and subsequent re-interventions. The baseline anatomical differences between our 2 cohorts provide strong validation for the appropriateness of our clinical indications. The criteria applied allowed successful identification of a “high-risk” subset of patients who were anatomically prone to persistent T2EL and subsequent sac expansion. The fact that the Coil+ group achieved a 0% sac expansion rate at 24 months demonstrates the clinical power of this proactive strategy, particularly given the significantly higher baseline risk due to larger IMA diameters and a greater number of patent LAs. Although the differences did not reach the level of statistical significance (*p* = 0.101) in the subgroup analysis of patients with anatomically high-risk features, the absence of sac expansion and secondary re-intervention in the Coil+ group may support the clinical value of prophylactic embolization in anatomically high-risk patients.

In the context of the current “reproducibility crisis” in biomedical research,^20)^ our results emphasize that standardized, science-based protocols may help overcome institutional volume differences. While low-volume centers are often cautioned regarding the learning curves associated with complex EVAR,^1)^ our experience shows that integrating objective anatomical assessment into the index procedure allows for the reproduction of high-level outcomes typically expected only from specialized centers.

Our multivariate analysis identified preoperative aneurysm diameter as an independent risk factor for sac expansion, a finding consistent with Laplace’s law and previous biomechanical assessments.^21)^ Consequently, prophylactic branch embolization should be strongly considered for patients with larger initial aneurysm diameters to ensure long-term sac stability and mitigate the risk of late enlargement.

Although pharmacological factors, such as antiplatelet or anticoagulant therapy, have been implicated in T2EL persistence,^1,21)^ no significant associations were observed in the present study. This suggests that mechanical exclusion of collateral pathways may play a more dominant role than systemic coagulation status in determining sac behavior.

Concerns have been raised that additional embolization procedures may prolong operative time and increase contrast exposure, potentially leading to renal impairment.^12)^ No significant differences in 1- or 2-year ΔeGFR were observed between the groups, suggesting that the additional embolization procedure did not result in measurable deterioration in renal function. This finding supports the procedural safety of prophylactic embolization with respect to renal function.

Notably, our institution achieved a near-100% follow-up rate, a stark contrast to the high attrition rates (approximately 60%) recently reported in the Western literature due to geographical^22)^ and logistical barriers.^23)^ This high adherence ensures patient safety, but also imposes a significant logistical burden on patients in rural regions where the centralization of cardiovascular care is progressing.^24)^ This “proactive strategy” to ensure early sac stability might potentially allow for reduced surveillance frequency^25)^ and thus could hold substantial social value by alleviating long-term burdens on both patients and the overstretched medical resources of small-scale regional centers.

The mean preoperative aneurysm diameter in the Coil+ group was 49.7 mm, reflecting the real-world clinical practice in our regional setting. In accordance with Japanese Circulation Society guidelines, EVAR was indicated for patients with smaller aneurysm diameters (≤50 mm) if they also presented with high-risk morphological features, such as a saccular configuration, rapid expansion over a short period, or concomitant expanding common iliac artery aneurysms requiring management.

### Study limitations

Several limitations should be acknowledged. First, this was a single-center retrospective study with a limited sample size. Second, the decision to perform embolization was not randomized and may have been influenced by anatomical or operator-related factors. Third, the intervals and modalities for imaging follow-up were not fully uniform, particularly in patients with renal dysfunction. Finally, the follow-up duration may not have been sufficient to capture late sac behavior in all patients. Further research with larger sample sizes and longer follow-up times is necessary to fully elucidate the implications of prophylactic branch embolization.

## Conclusion

Prophylactic branch embolization based on objective anatomical criteria appears to represent a reproducible strategy to achieve durable sac stability after EVAR. Given the limited evidence supporting secondary intervention for T2EL, a proactive approach focusing on anatomical exclusion at the index procedure may offer a rational pathway for optimizing long-term outcomes, even in low-volume centers.

## References

[1] Chaikof EL, Dalman RL, Eskandari MK, et al. The Society for Vascular Surgery practice guidelines on the care of patients with an abdominal aortic aneurysm. J Vasc Surg 2018; 67: 2–77.e2. 29268916 10.1016/j.jvs.2017.10.044

[2] Arko FR, Lee WA, Hill BB, et al. Aneurysm-related death: primary endpoint analysis for comparison of open and endovascular repair. J Vasc Surg 2002; 36: 297–304. 12170210 10.1067/mva.2002.126314

[3] Niklas N, Malec M, Gutowski P, et al. Effectiveness of inferior mesenteric artery embolization on type II endoleak-related complications after endovascular aortic repair (EVAR): systematic review and meta-analysis. J Clin Med 2022; 11: 5491. 36143138 10.3390/jcm11185491 PMC9506400

[4] Rayt HS, Sandford RM, Salem M, et al. Conservative management of type 2 endoleaks is not associated with increased risk of aneurysm rupture. Eur J Vasc Endovasc Surg 2009; 38: 718–23. 19767222 10.1016/j.ejvs.2009.08.006

[5] Seike Y, Matsuda H, Shimizu H, et al. Nationwide analysis of persistent type II endoleak and late outcomes of endovascular abdominal aortic aneurysm repair in Japan: a propensity-matched analysis. Circulation 2022; 145: 1056–66. 35209732 10.1161/CIRCULATIONAHA.121.056581 PMC8969842

[6] Koudounas G, Giannopoulos S, Charisis N, et al. Understanding type II endoleak: a harmless imaging finding or a silent threat? J Clin Med 2024; 13: 4250. 39064290 10.3390/jcm13144250 PMC11277561

[7] Vaddavalli VV, Zheng X, Mao J, et al. Outcomes associated with type II endoleaks after infrarenal endovascular aneurysm repair in the Vascular Quality Initiative linked to Medicare claims. J Vasc Surg 2025; 82: 810–8.e5. 40339998 10.1016/j.jvs.2025.04.061 PMC12354270

[8] Samura M, Morikage N, Mizoguchi T, et al. Effectiveness of embolization of inferior mesenteric artery to prevent type II endoleak following endovascular aneurysm repair: a review of the literature. Ann Vasc Dis 2018; 11: 259–64. 30402173 10.3400/avd.ra.18-00064 PMC6200615

[9] Ogino H, Iida O, Akutsu K, et al. JCS/JSCVS/JATS/JSVS 2020 guideline on diagnosis and treatment of aortic aneurysm and aortic dissection. Circ J 2023; 87: 1410–621. 37661428 10.1253/circj.CJ-22-0794

[10] Yu HYH, Lindström D, Wanhainen A, et al. Systematic review and meta-analysis of prophylactic aortic side branch embolization to prevent type II endoleaks. J Vasc Surg 2020; 72: 1783–92.e1. 32442608 10.1016/j.jvs.2020.05.020

[11] Li Q, Hou P. Sac embolization and side branch embolization for preventing type II endoleaks after endovascular aneurysm repair: a meta-analysis. J Endovasc Ther 2020; 27: 109–16. 31566053 10.1177/1526602819878411

[12] Saratzis A, Bown MJ. Renal injury after endovascular aneurysm repair: an overlooked entity. Eur J Vasc Endovasc Surg 2016; 51: 325–6. 26612598 10.1016/j.ejvs.2015.10.015

[13] Wu Y, Yin J, Hongpeng Z, et al. Systematic review and network meta-analysis of pre-emptive embolization of the aneurysm sac side branches and aneurysm sac coil embolization to improve the outcomes of endovascular aneurysm repair. Front Cardiovasc Med 2022; 9: 947809. 35935638 10.3389/fcvm.2022.947809 PMC9354492

[14] Lee SA, Kwon TW, Cho YP, et al. Long-term clinical outcomes of type 2 endoleak intervention and the crucial role of additional endoleak development after endovascular aneurysm repair: a single-center retrospective observational cohort study. Ann Surg Treat Res 2025; 109: 235–43. 41112351 10.4174/astr.2025.109.4.235 PMC12531634

[15] Samura M, Morikage N, Mizoguchi T, et al. Identification of anatomical risk factors for type II endoleak to guide selective inferior mesenteric artery embolization. Ann Vasc Surg 2018; 48: 166–73. 29275128 10.1016/j.avsg.2017.10.016

[16] Patel R, Sweeting MJ, Powell JT, et al. Endovascular versus open repair of abdominal aortic aneurysm in 15-years’ follow-up of the UK endovascular aneurysm repair trial 1 (EVAR trial 1): a randomised controlled trial. Lancet 2016; 388: 2366–74. 27743617 10.1016/S0140-6736(16)31135-7

[17] Takeuchi Y, Morikage N, Samura M, et al. Five-year follow-up of randomized clinical trial for pre-emptive inferior mesenteric artery embolization during endovascular aneurysm repair. J Vasc Surg 2024; 80: 693–701.e3. 38704104 10.1016/j.jvs.2024.04.058

[18] Ichihashi S, Takahara M, Fujimura N, et al. Editor’s choice – Multicentre randomised controlled trial to evaluate the efficacy of pre-emptive inferior mesenteric artery embolisation during endovascular aortic aneurysm repair on aneurysm sac change. Eur J Vasc Endovasc Surg 2025; 70: 219–26. 40252952 10.1016/j.ejvs.2025.04.028

[19] Ide T, Shimamura K, Shijo T, et al. Impact of patent lumbar arteries on aneurysm sac enlargement with type II endoleak after endovascular aneurysm repair. Eur J Vasc Endovasc Surg 2023; 66: 513–20. 37330200 10.1016/j.ejvs.2023.06.003

[20] Iqbal SA, Wallach JD, Khoury MJ, et al. Reproducible research practices and transparency across the biomedical literature. PLoS Biol 2016; 14: e1002333. 26726926 10.1371/journal.pbio.1002333 PMC4699702

[21] Bogdanovic M, Siika A, Lindquist Liljeqvist M, et al. Biomechanics and early sac regression after endovascular aneurysm repair of abdominal aortic aneurysm. JVS Vasc Sci 2023; 4: 100104. 37152845 10.1016/j.jvssci.2023.100104 PMC10160496

[22] Newton LE, Ponukumati A, Zwain G, et al. Imaging surveillance adherence after endovascular abdominal aortic aneurysm repair at Va hospitals. JAMA Netw Open 2025; 8: e256852. 40272801 10.1001/jamanetworkopen.2025.6852 PMC12022808

[23] Jarosinski MC, Olivere LA, Barnes JL, et al. Barriers and facilitators to endovascular aortic repair follow-up. JAMA Netw Open 2025; 8: e2546327. 41329483 10.1001/jamanetworkopen.2025.46327 PMC12673408

[24] Kanaoka K, Iwanaga Y, Nakai M, et al. Temporal trends and regional variations in cardiovascular care in Japan, 2010–2019. Int Heart J 2023; 64: 53–9. 36725073 10.1536/ihj.22-445

[25] Vanmaele A, Rastogi V, Oliveira-Pinto J, et al. Single centre evaluation of the proposal of the European Society for Vascular Surgery abdominal aortic aneurysm guidelines to stratify surveillance after endovascular aortic aneurysm repair. Eur J Vasc Endovasc Surg 2025; 69: 744–54. 39909310 10.1016/j.ejvs.2025.01.042

